# Antitumor activity of orally administered maitake α-glucan by stimulating antitumor immune response in murine tumor

**DOI:** 10.1371/journal.pone.0173621

**Published:** 2017-03-09

**Authors:** Yuki Masuda, Yoshiaki Nakayama, Akihiro Tanaka, Kenta Naito, Morichika Konishi

**Affiliations:** 1 Department of Microbial Chemistry, Kobe Pharmaceutical University, Kobe, Japan; 2 Research and Development Department, Yukiguni Maitake Co., Ltd., Niigata, Japan; Istituto Superiore di Sanità, ITALY

## Abstract

Maitake α-glucan, YM-2A, isolated from *Grifola frondosa*, has been characterized as a highly α-1,6-branched α-1,4 glucan. YM-2A has been shown to possess an anti-virus effect in mice; however, it does not directly inhibit growth of the virus *in vitro*, indicating that the anti-virus effect of YM-2A might be associated with modulation of the host immune system. In this study, we found that oral administration of YM-2A could inhibit tumor growth and improve survival rate in two distinct mouse models of colon-26 carcinoma and B16 melanoma. Orally administered YM-2A enhanced antitumor immune response by increasing INF-γ-expressing CD4^+^ and CD8^+^ cells in the spleen and INF-γ-expressing CD8^+^ cells in tumor-draining lymph nodes. *In vitro* study showed that YM-2A directly activated splenic CD11b^+^ myeloid cells, peritoneal macrophages and bone marrow-derived dendritic cells, but did not affect splenic CD11b^-^ lymphocytes or colon-26 tumor cells. YM-2A is more slowly digested by pancreatic α-amylase than are amylopectin and rabbit liver glycogen, and orally administered YM-2A enhanced the expression of MHC class II and CD86 on dendritic cells and the expression of MHC class II on macrophages in Peyer’s patches. Furthermore, in vitro stimulation of YM-2A increased the expression of pro-inflammatory cytokines in Peyer’s patch CD11c^+^ cells. These results suggest that orally administered YM-2A can activate dendritic cells and macrophages in Peyer’s patches, inducing systemic antitumor T-cell response. Thus, YM-2A might be a candidate for an oral therapeutic agent in cancer immunotherapy.

## Introduction

Cancer is the leading cause of death and disability worldwide. Cancer chemotherapy drugs kill rapidly growing cancer cells but can also harm normal cells, causing side effects throughout the body. As an alternative therapy, the potential ability of the immune system to recognize and reject tumors has been investigated for more than a century [1, 2]. Because of their relatively low toxicities, natural products have been studied for the development of new immunomodulatory agents.

Many studies have reported the immunomodulatory effects of polysaccharides isolated from mushrooms, fungi, yeast, algae, lichens, and plants. The maitake mushroom (*Grifola frondosa*) is a popular, edible mushroom in Japan, especially because its fruiting body can be artificially produced. We have reported previously that MD-Fraction, a highly purified, soluble β-(1,3)(1,6)-glucan derived from maitake mushrooms, induces antitumor activity by activating the host immune system [3, 4]. The maitake mushroom contains many bioactive molecules including polysaccharides and peptides, which have been reported to have various pharmacological effects, such as immunomodulation, anti-oxidation, and improvement of glucose and lipid metabolism [5–8]. Recently, the novel bioactive polysaccharide YM-2A was isolated from the maitake mushroom and characterized as a glycogen-like polysaccharide consisting of linear 4-linked α-D-Glc*p* residues substituted at position 6 with -α-D-Glc*p* branches [9]. Although oral administration of YM-2A has a preventive effect against influenza infection in mice, YM-2A does not directly inhibit growth of the virus *in vitro*. YM-2A protects immunocompromised mice from influenza infection and increases the titer of anti-virus antibody to the same extent as that seen in normal mice, suggesting that YM-2A could modulate the immune system in mice.

Glycogen is a major storage form of glucose found in many organisms ranging from bacteria to primates. The major functions of glycogen are to supply energy to muscle and to provide glucose to the circulation from the liver. In contrast to the traditional functions of glycogen, recent studies have reported that some glycogens have immunomodulatory activities [10–12]. In this study, we investigated whether oral administration of YM-2A exerts antitumor and immunomodulatory effects in a murine colon carcinoma model and an aggressive melanoma model. To identify the target cells of YM-2A, the direct immunomodulatory effects of YM-2A on various types of immune cells were evaluated. In addition, we studied how orally administered YM-2A can exhibit biological activity in the gastrointestinal tract.

## Materials and methods

### Preparation of YM-2A from maitake mushroom

YM-2A was prepared from maitake fruiting bodies (Yukiguni Maitake, Niigata, Japan) as previously described [9]. Lyophilized powder of maitake fruiting bodies was suspended in hot water (> 80°C) and then heat-extracted at 121°C for 30 min. The hot water extract was collected by centrifuge and the macromolecules were precipitated by the addition of ethanol to a concentration of 50%. The precipitate was dissolved in distilled water and then the supernatant was collected by the addition of ethanol (25% final concentration). The precipitate (YM-2A) was collected by the addition of ethanol (42% final concentration), dissolved in distilled water, freeze-dried, and kept in desiccators at room temperature (25°C). For the experiments, YM-2A powder was dissolved in distilled water to a final concentration of 25 mg/ml and treated at 100°C for 5 min, then stored at 4°C until use. The weight-average molecular weight of YM-2A was calculated to be 8.51 × 10^6^ Da by high-performance size-exclusion chromatography (HPSEC) analysis with a multi-angle laser light scattering detector (MALS; DAWN HELEOS, Wyatt Technology, Santa Barbara, CA) [9]. ^1^H and ^13^C NMR and methylation analysis revealed that YM-2A is a glycogen-like polysaccharide consisting of linear 4-linked α-D-Gluc*p* residues substituted at position 6 with -α-D-Gluc*p* branches. ^13^C NMR detected peaks at 100.6, 78.0 and 61.3ppm (S1 Fig). It has been reported that the chemical shift of the anomeric carbon C-1 in both β-1,4-D-glucan and β-1,6-D-glucan is about 103 ppm, while the shift of C-1 in α-1,4-D-glucan is around 100.6 ppm [13, 14]. Thus the peak at 100.6 ppm suggested C-1 in α-1,4-D-glucan by chemical shift position, and the peak at 78.0 and 61.3ppm suggested C-4 and C-6, respectively. We notice that the peak associated with β-glucan was not observed, suggesting that YM-2A sample using in this study has almost no contamination of maitake β-glucan, MD-Fraction.

### Mice

Female BALB/c, C57BL/6 and DBA/2 mice were purchased from CLEA Japan (Higashiyama, Japan), and 5–8-week-old mice were used in the study. This study was carried out under the guidelines of the Animal Care and Use Committee at the Kobe Pharmaceutical University. The protocol was approved by the Committee on the Ethics of Animal Experiments of the Kobe Pharmaceutical University (Permit Number: 2016–39). At the end of the experiments, mice were sacrificed by cervical dislocation under isoflurane anesthesia and all efforts were made to minimize suffering.

### Cell preparation and culturing

Spleen single-cell suspensions were prepared by filtration through 70 μm nylon strainers (BD Biosciences), and CD11b^+^/CD11b^-^ cells were separated by using CD11b MicroBeads (Miltenyi Biotec, Bergisch Gladbach, Germany). Resident peritoneal macrophages were harvested by peritoneal lavage with PBS, and the adherent cells were enriched by plastic adherence in complete RPMI (RPMI-1640 supplemented with 10% FBS, 0.03 mg/ml L-glutamine, 100 units/ml penicillin, and 100 μg/ml streptomycin) for 2 h and used as macrophages. Bone marrow–derived dendritic cells (DCs) were grown in complete RPMI supplemented with murine GM-CSF (20 ng/ml) and IL-4 (10 ng/ml), as described previously [4]. DCs were harvested and analyzed on day 7–8, and 85% of the cells expressing CD11c were used. Peyer's patches were recovered and digested in the presence of Liberase^TM^ (Roche, Indianapolis, IN) and DNase I (Sigma-Aldrich, St Louis, MO) for 30 min. This preparation was then passed through a cell strainer to make single cell suspensions. Enrichment for DC populations was then performed by positive selection on CD11c-MACS beads (Miltenyi Biotec). Whole spleen cells and CD11b^-^ cells (5 × 10^6^ cells/ml), CD11b^+^ cells (2 × 10^6^ cells/ml), macrophages, bone marrow-derived DCs (1 × 10^6^ cells/ml) and peyer’s patch CD11c^+^ cells (5 × 10^5^ cells/ml) were stimulated with YM-2A at various concentrations for 24 h. After incubation, the relative number of viable cells in each well was measured with WST-8 reagent using the Cell Count Reagent SF (Nacalai Tesque, Kyoto, Japan). TNF-α and IL-12 levels in the supernatants were determined using ELISA kits, according to the manufacturer’s protocol (PeproTech, Rocky Hill, NJ, USA). Real-time qPCR was used to examine mRNA expression levels of various cytokines. RNA was purified using RNeasy (Qiagen, Hilden, Germany) before first-strand cDNA synthesis using the ReverTra Ace qPCR RT kit (Toyobo, Osaka, Japan). qPCR using Thunderbird SYBR qPCR mix (Toyobo) was performed using primers for 18S, IL-12p40, IL-12p35, TNF-α, IL-1β, and IL-6, as described previously [15].

Glucose, maltose, maltotriose, α-cyclodextrin, and starch were purchased from Nacalai Tesque (Kyoto, Japan). Oyster glycogen was purchased from Wako Pure Chemical (Osaka, Japan), amylopectin was from MP Biomedicals, Inc. (Solon, OH), and slipper limpet (Type VIII) and rabbit river glycogen (Type III) were from Sigma-Aldrich (St Louis, MO).

### Treatment of α-amylase from human pancreas

α-Amylase from human pancreas was purchased from Sigma-Aldrich (St Louis, MO). Glucan (10 mg/ml) was incubated with 0.2 U/ml of α-amylase at 20°C. At the end of the incubation period, the mixture was heated at 100°C for 10 min to inactivate the enzyme. The digested glucans were filtered with a 0.45 μM membrane (Toyo Roshi Kaisha, Ltd., Tokyo, Japan), applied to HPSEC using an SB-806M HQ Shodex column (Showa Denko, Tokyo, Japan) and monitored with a differential refractive index detector. Elusion was carried out with 0.1 M NaNO_3_ at a flow rate of 1 ml/min.

### In vivo mouse models

Treatment groups were control, pre-treatment of YM-2A (2.5 or 5 mg/day), and post-treatment of YM-2A (5 mg/day). Mice were orally administered either distilled water (as the control) or YM-2A for 5 days/week from day 0 (pre-treatment) or day 8 (post-treatment). BALB/c or C57BL/6 mice were inoculated with colon-26 carcinoma cells (1 × 10^5^ cells/mouse) or B16 melanoma cells (2 × 10^5^ cells/mouse), respectively, by subcutaneous (sc) injection on day 7. The tumor volume was calculated using the formula Tumor volume (cm^3^) = (longest diameter × shortest diameter^2^)/2. Mice were sacrificed when their tumor volumes were more than 2.25 cm^3^. To investigate the *in vivo* immunomodulatory effect of YM-2A (pre-treatment, 5 mg/day), animals were sacrificed on day 14 after tumor inoculation and the spleen, tumor-draining lymph node (TDLN), Peyer’s patch (PP), and tumor were resected.

### Flow cytometry

Spleen, TDLN and PP single-cell suspensions were blocked with Fc receptor blocking mAb and stained with mAb including CD4, CD8, CD49b, CD3, B220, CD11c, CD80, CD86, I-A/I-E, Gr-1 (BD PharMingen, San Diego, CA) or F4/80 (AbD Serotec, Kidlington, UK) for 20 min at 4°C, then washed and analyzed using a flow cytometer (FACSAria; BD Biosciences). For the analysis of BrdU incorporation, macrophages or DCs were stimulated with YM-2A in presence of 10 μM BrdU (BrdU Flow Kit, BD PharMingen) for 24 h, and cells were stained for cell surface markers and BrdU using the BrdU Flow Kit (BD Biosciences). For intracellular staining of IFN-γ and IL-4, cells were cultured for 4 h with PMA (25 ng/ml) and ionomycin (1 μg/ml), and cytokine release was prevented by treatment with Golgi-stop (BD PharMingen). Following surface staining of CD4 or CD8, cells were fixed using the Cytofix/Cytoperm kit (BD PharMingen) and stained with mAb including IFN-γ or IL-4 (BD PharMingen).

### Statistical analysis

Data were analyzed using Prism software (GraphPad Software, Inc). Data presented are expressed as the mean ± standard error (SE). One-way ANOVA with the Tukey’s post hoc test was used for analysis of multiple groups, and Student’s t-test was used to compare the two groups. Tumor growth data were analyzed using a two-way ANOVA. Kaplan-Meier survival curves of mice in the tumor studies were analyzed by the log rank survival test. Differences were considered statistically significant at p < 0.05.

## Results

### YM-2A provides therapeutic benefit in colon-26-bearing mice

**Fig 1:**
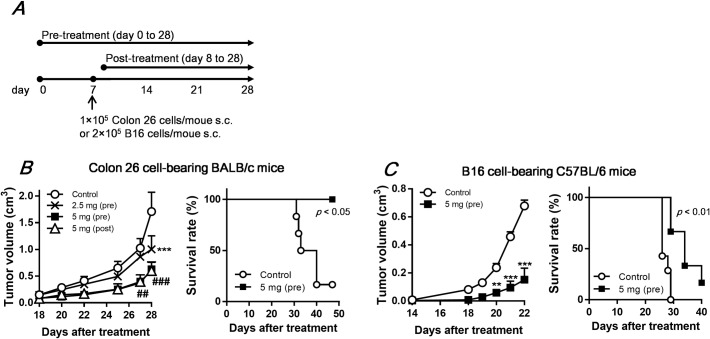
YM-2A provides therapeutic benefit in colon-26 tumor-bearing mice. (A) Experimental scheme. YM-2A (2.5 mg or 5 mg/mouse) was orally administered (daily, 5 days/week) from day 0 (pre-treatment) or day 8 (post-treatment). (B and C, left) Tumor volume (n = 5 for colon26 model, n = 6~7 for B16 model) represents the means ± S.E. of two separate experiments. **p < 0.01 and ***p < 0.001, compared with control. ##*p* < 0.01 and ###*p* < 0.001 indicate significant differences both in 5 mg (pre) vs control and 5 mg (post) vs control. (B and C, right) Mice were euthanized when subcutaneous tumors reached 2.25 cm^3^. Survival rates are represented using Kaplan–Meier curves.

Although oral administration of YM-2A has a preventive effect against influenza infection in mice, YM-2A does not directly inhibit growth of the virus *in vitro* [9], suggesting that YM-2A could modulate the immune system in mice. This result prompted us to evaluate the antitumor and immunomodulatory properties of YM-2A *in vivo* and *in vitro*. First, we examined the preventive and therapeutic effects of YM-2A on subcutaneous colon-26 tumor growth in BALB/c mice (Fig 1A). The pre-administration of YM-2A inhibited colon-26 tumor growth in a dose-dependent manner (Fig 1B). Pre-administration of YM-2A (5 mg/mouse) not only decreased tumor growth but also increased survival of mice (Fig 1B). Similar to the inhibition from pre-administration of YM-2A, post-administration of YM-2A (5 mg/mouse) significantly inhibited colon-26 tumor growth, suggesting that YM-2A has both preventive and therapeutic effects on tumor growth (Fig 1B). We next examined effect of YM-2A on tumor growth in vivo using another mouse models, B16 melanoma-bearing C57BL/6 mice (Fig 1A). The pre-administration of YM-2A (5 mg/mouse) significantly inhibited tumor growth and increased survival in B16 melanoma model (Fig 1C). These finding suggest that oral administration of YM-2A is an effective therapy for cancer.

### YM-2A directly activates macrophages and DCs, but not colon-26 tumor cells

**Fig 2:**
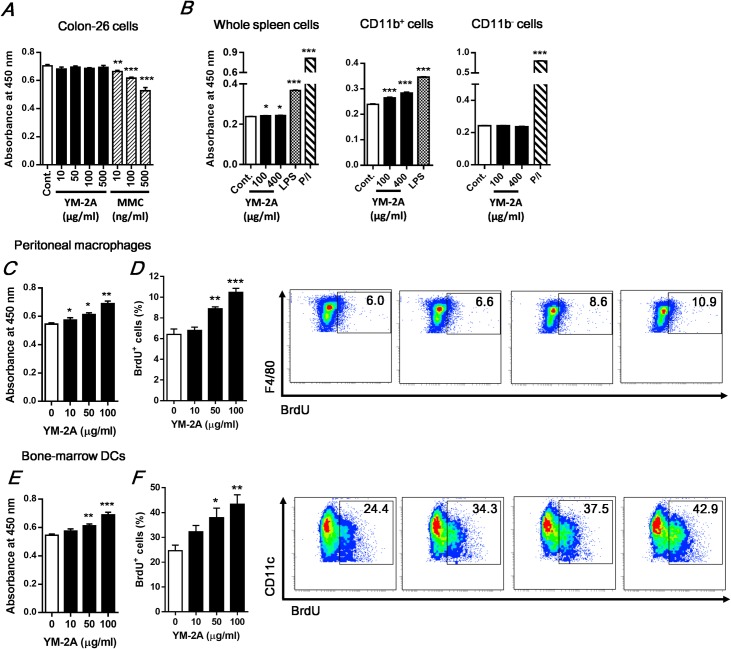
YM-2A directly enhances proliferation from macrophages and DCs. Colon-26 tumor cells (A), spleen cells (B), peritoneal macrophages (C and D), and bone marrow DCs (E and F) were incubated with YM-2A or indicated compounds for 24 h. (C and E) After incubation, cell proliferation was measured using WST-8 assay. (D and F) For BrdU Assay, cells were incubated with YM-2A in presence of BrdU (10 μM). Representative plots (right) and summarized data (left) were shown as the percentage of cells incorporating BrdU. The data presented are representative of two independent experiments. *p < 0.05, **p < 0.01 and ***p < 0.001, compared with control or 0 μg/ml of YM-2A.

As oral administration of YM-2A had a significant antitumor effect in mice, we further investigated whether tumor growth inhibition was induced by the direct cytotoxicity of YM-2A. After the incubation of colon-26 tumor cells with YM-2A *in vitro*, the cell numbers were assessed by WST-8 assay. Fig 2A shows that YM-2A did not affect the cell numbers of colon-26 tumor cells, even at the high concentration of 500 μg/ml, whereas the DNA-damaging agent mitomycin C significantly decreased the cell numbers in a dose-dependent manner. This result indicates that YM-2A does not directly inhibit colon-26 tumor cell growth.

We next investigated whether YM-2A directly stimulates immune cells *in vitro* by WST-8 assay to measure cell proliferation. Fig 2B shows that YM-2A very slightly but significantly increased cell proliferation of whole spleen cells. To investigate the type of immune cells involved in the activity of YM-2A, splenic CD11b^+^ and CD11b^-^ cells were separated by the magnetic cell separation system using anti-CD11b microbeads and stimulated with YM-2A, LPS (as a positive control for CD11b^+^ cells) or PMA/Ionomycin (as a positive control for CD11b^-^ cells). As shown in Fig 2B, YM-2A increased the cell number of CD11b^+^ cells, but not CD11b^-^ cells. These results suggest that CD11b^+^ myeloid cells play a major role in the recognition of YM-2A, but CD11b^-^ cells, containing primarily lymphocytes, do not. Splenic CD11b^+^ cells contain many distinct cell types, such as macrophages and dendritic cells (DCs), which are important in the initiation of immune response. Therefore, we investigated the proliferation activity of YM-2A on peritoneal resident macrophages and bone marrow-derived DCs using WST-8 Assay and BrdU incorporation. Relative values to the number of viable cells were measured by WST-8 assay, and their DNA synthesis was assessed by BrdU incorporation assay. As expected, both WST-8 Assay and BrdU incorporation assay showed that YM-2A increased cell proliferation of these cells in a dose-dependent manner (Fig 2C–2F).

**Fig 3:**
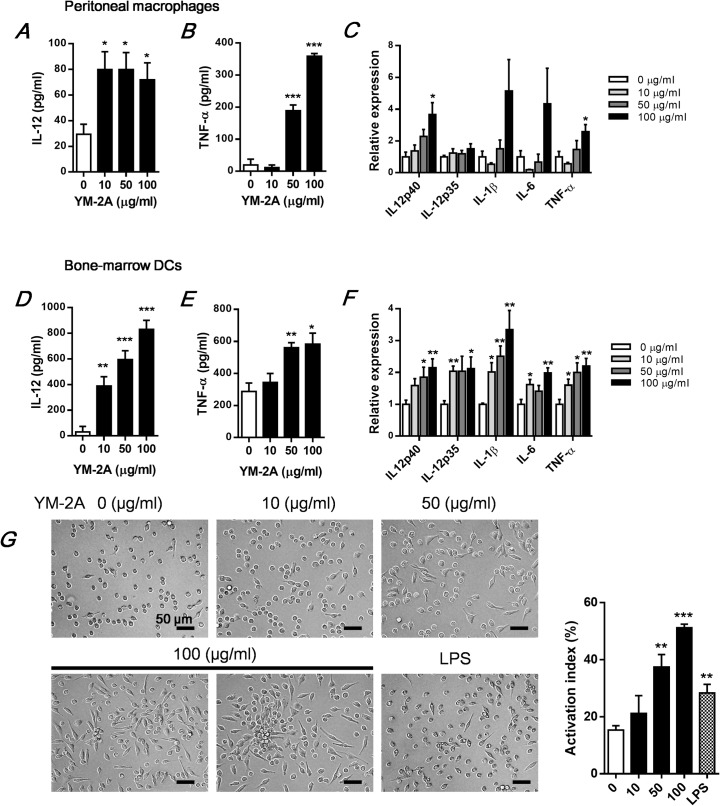
YM-2A increases various cytokine production from macrophages and DCs. Peritoneal macrophages (A-C), and bone marrow DCs (D-F) were incubated with YM-2A for 24 h. After incubation, TNF-α or IL-12 levels in the collected supernatants were measured by ELISA (A, B, D and E) and mRNA expression levels of cytokines were determined by real-time PCR (C and F). The relative expression level was normalized to the expression level with 0 μg/ml control. (G) Morphological change in macrophage. Morphology of macrophage visualized by optical microscopy (×400) (G, left panels). The activation index percentage was expressed as the number of cells with activated morphology relative to the total number of cells, quantified in 3–4 random fields (number of total cells >100) (G, right panels). The data presented are representative of two independent experiments. *p < 0.05, **p < 0.01 and ***p < 0.001, compared with 0 μg/ml control.

Next, we investigated whether YM-2A affect production of pro-inflammatory cytokines (TNF-α, IL-12, IL-1β and IL-6) by macrophages and DCs. YM-2A increased TNF-α and IL-12 secretion from macrophages and DCs (Fig 3A, 3B, 3D and 3E). In addition, IL-12p40 and TNF-α mRNA in macrophages, and IL-12p40, IL-12p35, IL-1β, IL-6 and TNF-α mRNA levels in DCs were significantly increased by YM-2A stimulation (Fig 3C and 3F). We also found that treatment with YM-2A promoted the morphological changes in macrophages indicative of cellular activation (Fig 3G). In the unstimulated cells, the cell morphology generally showed a round form, whereas YM-2A-treated macrophages had changed to an irregular form with accelerated adherence (i.e. cell spreading and adhering to the culture dish) (Fig 3G, left). These morphological changes are known to be a signature event of activated macrophages [16]. The percentage of activated macrophages to total cells, an index reflecting the extent of cell spreading, was dose-dependently increased by YM-2A (Fig 3G, right). These results suggest that YM-2A directly activates antigen-presenting cells, such as macrophages and DCs, and induces the production of pro-inflammatory cytokines.

### YM-2A is the most effective agent among various carbohydrates to activate macrophages

**Fig 4:**
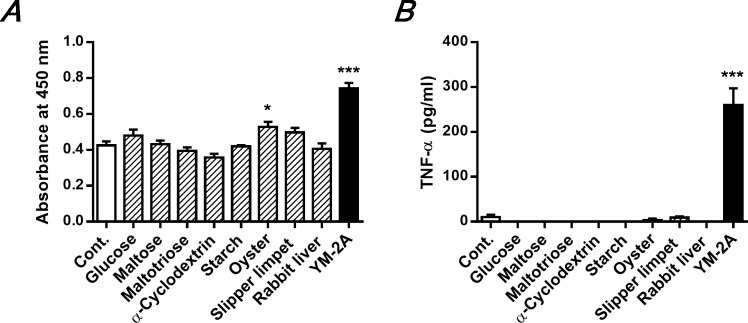
Immunomodulatory effect of YM-2A on macrophages is more effective than that of various carbohydrates. Peritoneal macrophages were incubated with YM-2A or indicated compounds at a concentration of 100 μg/mL for 24h. After incubation, cell proliferation (A) and TNF-α levels (B) in the supernatants were measured. *p < 0.05 and ***p < 0.001, compared with control.

The previous study characterized YM-2A as a glycogen-like polysaccharide consisting of linear 4-linked α-D-Glc*p* residues substituted at position 6 with -α-D-Glc*p* branches [9]. Recent reports have demonstrated that natural and synthetic glycogens exert immunomodulatory effects, but some glycogens have little or no activity [17–19]. Therefore, we compared the immunomodulatory activity of YM-2A on murine peritoneal macrophages with various carbohydrates (Fig 4). Mono- and oligosaccharides (glucose, maltose, maltotriose, and α-cyclodextrin) did not influence proliferation of macrophages. In contrast, oyster glycogen (*p* = 0.03) and slipper limpet glycogen (*p* = 0.07) slightly promoted proliferation of macrophages, but starch (*p* = 0.8) and rabbit liver glycogen (*p* = 0.6) did not. These results were consistent with a previous report using RAW264.7 cells [17]. Only YM-2A significantly induced both proliferation and TNF-α production from murine peritoneal macrophages, indicating that YM-2A is more effective to activate the immune system than are various other glycogens used in this experiment.

### Oral administration of YM-2A modulates systemic immune responses, resulting in antitumor T-helper (Th)-1 and cytotoxic T-cell (CTL) priming

**Fig 5:**
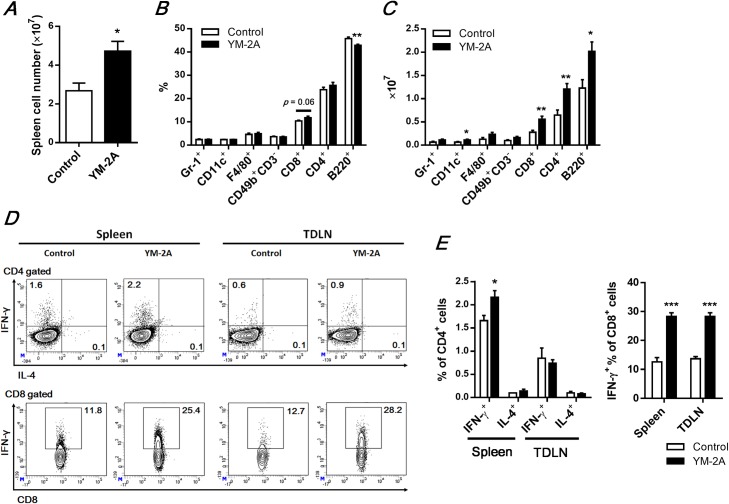
Orally administered YM-2A enhances systemic antitumor immunity. On day 14 after colon-26 inoculation in BALB/c mice, splenocytes were stained with specific antibodies and analyzed by flow cytometry. Total spleen cell number (A), percentages (B) and numbers (C) of each cell in a whole-spleen cell suspension are shown. (D and E) The expression of IFN-γ in gated CD4^+^ or CD8^+^ T cells in the spleen, and tumor draining lymph node (TDLN). Cells were cultured for 4 h with PMA (25 ng/ml) and ionomycin (1 μg/ml) in presence of Golgi-stop and then stained for intracellular IFN-γ or IL-4 production. Representative plots (D) and summarized data (E) of IFN-γ-expressing CD4^+^ and CD8^+^ T cells. *p < 0.05, **p < 0.01, and ***p < 0.001, compared with the control.

Since YM-2A showed a direct immunomodulatory effect *in vitro*, we next investigated how YM-2A affected the systemic immune system *in vivo*. The spleen is the largest secondary immune organ containing about one-fourth of the body’s lymphocytes and initiates immune responses to blood-borne antigens [20]. Oral administration of YM-2A (5 mg/mouse) significantly increased spleen weight (data not shown) and total number of spleen cells (Fig 5A) in colon-26 tumor-bearing mice, suggesting that orally administered YM-2A can enhance the systemic immune system. YM-2A increased the percentages of CD8^+^ cells in spleen cells compared with percentages in the control mice (*p* = 0.06), while it decreased the percentage of B cells (B220^+^) and did not affect the percentages of granulocytes (Gr-1^+^), DCs (CD11c^+^), macrophages (F4/80^+^), NK cells (CD49b^+^CD3^-^), or CD4^+^ cells in spleen cells (Fig 5B). As total number of spleen cells were increased by YM-2A, we calculated the numbers of each immune cell subtype. As shown in Fig 5C, YM-2A significantly increased the cell numbers of splenic DCs (1.74-fold), CD4^+^ T cells (1.87-fold) and CD8^+^ T cells (2.0-fold) and B cells (1.64-fold) compared with the control group. These results suggest that YM-2A may induce cellular rather than humoral immunity.

Antitumor immunity is initiated by antigen-presenting cells that capture tumor antigens from tumor cells and induce the CD4^+^ Th-1 cell/CD8^+^ CTL response, and they produce the antitumor cytokine IFN-γ [21]. Since YM-2A directly activates antigen-presenting cells *in vitro* and increased the numbers of splenic DCs and T cells *in vivo*, we next assessed whether oral administration of YM-2A can induce IFN-γ expression by CD4^+^ and CD8^+^ T cells in tumor-bearing mice. Intracellular cytokine staining showed that YM-2A significantly increased the percentage of IFN-γ-expressing cells in splenic CD4^+^ and CD8^+^ T cells, but not IL-4-expressing cells in CD4^+^ T cells (Fig 5D and 5E). As tumor-draining lymph nodes (TDLNs) are the primary site for the antitumor immune response, we also examined antitumor T-cell response in TDLNs. Neither the percentage of CD4^+^ cells in TDLN (data not shown) nor the percentage of IFN-γ^+^ and IL-4^+^ cells in CD4^+^ cells were altered by oral administration of YM-2A, whereas both the percentage of CD8^+^ cells (data not shown) and the percentage of IFN-γ^+^ cells in CD8^+^ cells were significantly increased (Fig 5D and 5E). These data combined with the observed enhanced production of IL-12 by DCs in response to stimulation of YM-2A (Fig 3D and 3F) indicate that YM-2A enhances Th1 polarization and induces antitumor CD8^+^ T cell development.

### Oral administration of YM-2A activates macrophages and DCs in the gut-associated lymphoid tissue (GALT)

**Fig 6:**
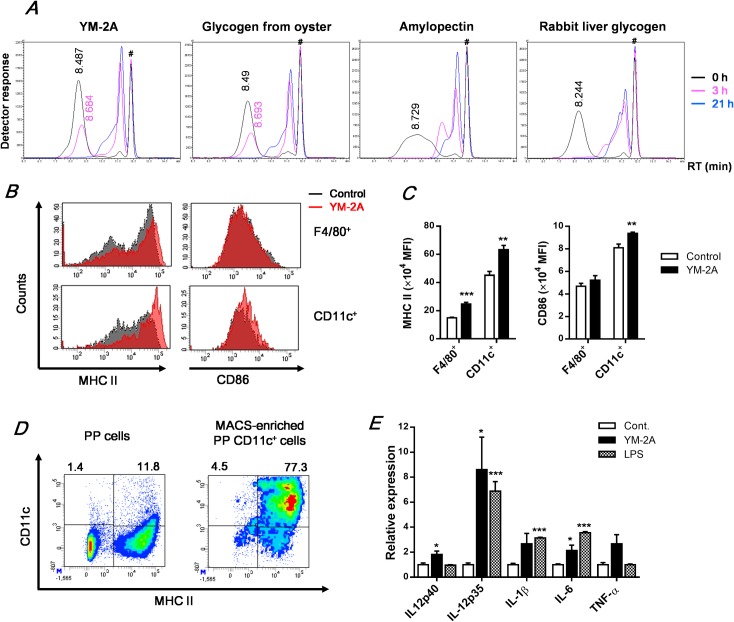
YM-2A is resistant to digestive enzymes and orally administered YM-2A activates antigen-presenting cells in GALTs. (A) After treatment with pancreatic α-amylase, glycogens were analyzed using HPLC. #Negative solvent peak (11.84 min). (B) On day 14 after colon-26 inoculation in BALB/c mice, Peyer’s patch cells were stained with specific antibodies. Histograms showing expression level of MHC class II and CD86 were measured by flow cytometry. (C) Mean fluorescence intensity (MFI) expressed by macrophages (F4/80^+^) or DCs (CD11c^+^) was analyzed. (D) Whole Peyer’s patch cells and CD11c^+^ MACS column-enriched Peyer’s patch CD11c^+^ cells were stained with anti-MHC class II and CD11c. (E) MACS-enriched Peyer’s patch CD11c^+^ cells were incubated with YM-2A (100 μg/ml) or LPS (1 μg/ml) for 24 h. After incubation, mRNA expression levels of cytokines were determined by real-time PCR. The relative expression level was normalized to the expression level with the control. *p < 0.05, **p < 0.01, and ***p < 0.001, compared with the control.

Because YM-2A is an α-glucan consisting of α-1,4 and α-1,6 linkages, orally administered YM-2A is considered to be digested by pancreatic α-amylase, maltase, isomaltase, and glucoamylase acting in the intestinal surface. Therefore, we tested the degradation of YM-2A treated with pancreatic α-amylase and compared digestion profiles of YM-2A and other carbohydrates by HPLC analysis (Fig 6A). Retention time (RT) of YM-2A at 0 h and 3 h was 8.487 min and 8.684 min, respectively. A similar result, that glycogen from oyster was slowly degraded by pancreatic α-amylase, was observed with RT of 8.49 min and 8.693 min at 0 h and 3 h, respectively. At 21 h after treatment, macromolecules of both YM-2A and oyster glycogen were completely degraded. On the other hand, amylopectin (RT 8.729 min at 0 h) and rabbit liver glycogen (RT 8.244 min at 0 h) were immediately degraded and no macromolecules remain at 3 h after treatment of pancreatic α-amylase. These results suggest that some glycogens from natural sources are more resistant to small intestinal enzymes than are amylopectin and liver glycogen.

It has been reported that enzymatically synthesized glycogen activates primary cultured Peyer’s patch cells [12]. Peyer's patches are lymphoid tissues found in the wall of the small intestine and part of the GALTs. Since YM-2A was resistant to pancreatic α-amylase, orally administered YM-2A macromolecules may be able to interact with the GALTs. To investigate whether orally administered YM-2A activates the GALTs, we analyzed the expression of activation markers, MHC class II and CD86, on macrophages and DCs in the Peyer’s patch. Fig 6B and 6C show that oral administration of YM-2A enhanced the expression of MHC class II and CD86 on DCs (CD11c^+^), while it enhanced only the expression of MHC class II on macrophages (F4/80^+^). To investigate whether YM-2A can directly activate Peyer’s patch DCs, we enriched CD11c^+^ DCs from Peyer’s patch cells by MACS system. The proportion of CD11c^+^ cells in whole Peyer’s patch cells was 13.2%, while that in MACS-enriched Peyer’s patch CD11c^+^ cells was 81.8% (Fig 6D). In vitro stimulation of YM-2A significantly increased the expression of IL-12p40, IL-12p35 and IL-6 in MACS-enriched Peyer’s patch CD11c^+^ cells (Fig 6E). Taken together, these results suggest that orally administered YM-2A is resistant to digestive enzymes and activates antigen-presenting cells in the GALTs.

## Discussion

Since antitumor immunotherapy using immunomodulatory agents has been known to be beneficial with high efficacy and safety, market of immunotherapy is growing worldwide [1, 2]. Especially, polysaccharides isolated from natural sources are used for clinical trial and therapy in cancer patients [22–24]. Recently, some α-glucans consisting of highly α-(1→6)-branched α-(1→4)-glucan, glycogen-like structures, have been reported to possess immunomodulatory activity [25–27]. In this study, we demonstrated that maitake α-glucan, YM-2A, significantly inhibited tumor growth by enhancing the immune system.

Oral administration of YM-2A significantly delayed tumor growth and increased survival in two distinct mouse models (Fig 1). We found that YM-2A did not directly inhibit tumor cell growth, but increased cell proliferation of antigen-presenting cells, such as macrophages and DCs, *in vitro* (Fig 2). YM-2A promoted production of IL-12 and TNF-α from both macrophages and DCs (Fig 3A, 3B, 3D and 3E). In addition, YM-2A up-regulated mRNA expression of IL-12p40 and TNF-α in macrophages, and IL-12p40, IL-12p35, IL-1β, IL-6 and TNF-α in DCs (Fig 3C and 3F), indicating that YM-2A treatment induced a strong inflammatory phenotype in these cells. IL-12 was previously reported to mediate the expression of other pro-inflammatory cytokines, TNF-α, IL-1β, and IL-6, in DCs [28], and IL-12 produced by antigen-presenting cells induces the production of IFN-γ by T cells, and favor the Th1 response that bridges innate and adaptive immunity [29, 30]. In a colon-26 tumor model, oral administration of YM-2A induced IFN-γ expression by CD4^+^ T cells in the spleen, and CD8^+^ T cells in the spleen and TDLNs (Fig 5D and 5E). These results indicate that YM-2A may be recognized by antigen-presenting cells and induce the production of the Th1 polarizing cytokine IL-12 by these cells, and subsequently induce antitumor T-cell responses. However, it remains unclear how YM-2A activates macrophages and DCs. Some glycogens (e.g. enzymatically synthesized glycogen, α-glucan from *Pseudallescheria boydii*, α-glucan from *Lentinula edodes*, and Mycobacterial α-glucan) were reported to activate macrophages through Toll-like receptor (TLR) 2, TLR 4, or dendritic cell-specific ICAM-3-grabbing nonintegrin (DC-SIGN) [18, 25, 27, 31]. Further studies are required to determine the molecular mechanism of the effect of YM-2A on macrophages and DCs.

Comparing the immunomodulatory activity of YM-2A and various carbohydrates on peritoneal macrophages, only YM-2A induced both proliferation and TNF-α production (Figs 2 and 3). On the other hand, oyster glycogen slightly promoted proliferation of macrophages, but mono- and oligosaccharide, starch (contains amylopectin), and rabbit liver glycogen did not. Despite rabbit liver glycogen having a high molecular weight and consisting of α-(1→6)-branched α-(1→4)-glucan similar in structure to YM-2A, it does not possess immunomodulatory activity. Further structural analysis is needed to elucidate the structure-activity relationship. As low-molecular-weight carbohydrates do not possess immunomodulatory activity, YM-2A might lose its activity after complete digestion with digestive enzymes. Interestingly, YM-2A and oyster glycogen are resistant to pancreatic α-amylase, unlike amylopectin and rabbit liver glycogen (Fig 6A), consistent with their activity pattern in macrophages. Taken together, these results indicate that YM-2A is more effective than various glycogens in activating the immune system *in vitro* and *in vivo*. Orally administered enzymatically synthesized glycogen has been reported to stimulate GALT cells and/or affect the composition of intestinal commensal microbiota [12, 32, 33]. Consistent with this, orally administered YM-2A could activate macrophages and DCs in GALT (Fig 6B and 6C). Furthermore, YM-2A directly up-regulated IL-12p40, IL-12p35 and IL-6 mRNA expression in Peyer’s patch CD11c^+^ cells (Fig 6E). YM-2A was resistant to pancreatic α-amylase for 3 h, but was completely digested after 21 h of treatment, suggesting that YM-2A could stimulate GALT cells within several hours of oral administration, and then might lose its activity upon digestion. In addition, YM-2A is completely resistant to salivary α-amylase (data not shown), supporting its clinical efficacy by oral administration. Another possibility, the influence of YM-2A on the intestinal commensal microbiota, should be investigated further.

In summary, our results provide evidence that maitake α-glucan YM-2A is a novel antitumor therapeutic agent, which can directly activate macrophages and DCs and induce Th1/CTL immune responses to inhibit tumor cell growth. Since YM-2A is more resistant to digestive enzymes than are amylopectin and rabbit liver glycogen, orally administered YM-2A can activate macrophages and DCs in GALTs. The immunomodulatory effect of YM-2A will make it a promising candidate as an oral therapeutic agent in the translational and clinical research of antitumor immunotherapy.

## Supporting information

S1 Fig ^13^C NMR patterns of YM-2A. Added 600μL D2O to 30.1 mg of sample and dissolved at 90℃, and measured. The peak at 100.6 ppm suggested C-1 in α-1,4-D-glucan by chemical shift position, and the peak at 78.0 and 61.3ppm suggested C-4 and C-6, respectively.
(TIF)
