# Supplementary figures and images for: Antitumor activity of orally administered maitake α-glucan by stimulating antitumor immune response in murine tumor
Source: PLoS One. 2017 Mar 9;12(3):e0173621. doi: 10.1371/journal.pone.0173621 (PMC5344464; doi:10.1371/journal.pone.0173621)

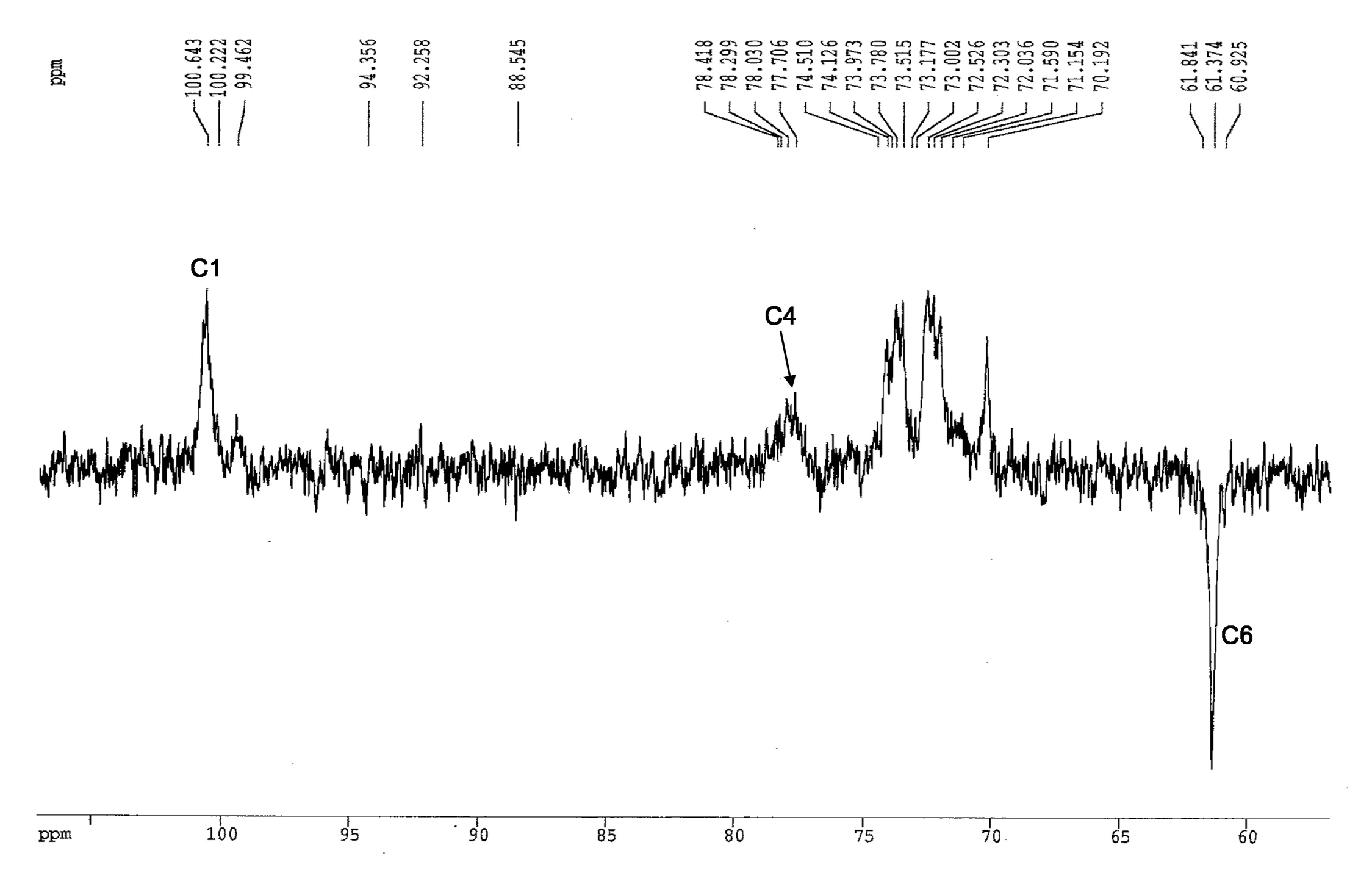

Supplement: S1 Fig — Added 600μL D2O to 30.1 mg of sample and dissolved at 90℃, and measured. The peak at 100.6 ppm suggested C-1 in α-1,4-D-glucan by chemical shift position, and the peak at 78.0 and 61.3ppm suggested C-4 and C-6, respectively. (TIF) [file pone.0173621.s001.tif]
